# Adherence to the Dietary Approaches to Stop Hypertension Dietary Pattern and Risk of Abdominal Aortic Aneurysm: Results From the ARIC Study

**DOI:** 10.1161/JAHA.118.009340

**Published:** 2018-11-02

**Authors:** Bernhard Haring, Elizabeth Selvin, Xintong He, Josef Coresh, Lyn M. Steffen, Aaron R. Folsom, Weihong Tang, Casey M. Rebholz

**Affiliations:** ^1^ Department of Internal Medicine I University of Würzburg Bavaria Germany; ^2^ Department of Epidemiology Johns Hopkins Bloomberg School of Public Health Baltimore MD; ^3^ Division of Epidemiology and Community Health University of Minnesota School of Public Health Minneapolis MN; ^4^ Welch Center for Prevention Epidemiology, and Clinical Research Johns Hopkins University Baltimore MD

**Keywords:** aneurysm, diet, Dietary Approaches to Stop Hypertension, Diet and Nutrition, Epidemiology, Vascular Disease, Aneurysm

## Abstract

**Background:**

The role of a healthy dietary pattern in the prevention of abdominal aortic aneurysms (AAA) is unknown. We aimed to evaluate the relationship between adherence to a Dietary Approaches To Stop Hypertension‐style dietary pattern and the risk of incident AAAs.

**Methods and Results:**

Dietary intake was assessed via a 66‐item food frequency questionnaire at baseline (1987–1989) and at visit 3 (1993–1995) in 13 496 participants enrolled in the ARIC (Atherosclerosis Risk in Communities) study without clinical AAA (mean age, 54 years). A dietary scoring index based on food times was constructed to assess self‐reported adherence to a dietary approaches to stop hypertension‐style dietary pattern. Participants were followed for incident clinical AAAs using hospital discharge diagnoses, Medicare inpatient and outpatient diagnoses, or death certificates through December 31, 2011. Cox proportional hazards models with covariate adjustment were used to estimate hazard ratios with 95% confidence intervals. During a median follow‐up of 23 years, there were 517 incident AAA cases. Individuals with a Dietary Approaches To Stop Hypertension‐style diet score in the highest quintile had a 40% lower risk of hospitalization for AAA than those in the lowest quintile (hazard ratio_Q5 vs Q1_: 0.60; 95% confidence intervals: 0.44, 0.83; *P*_trend_=0.002). In detailed analyses, higher consumption of fruits, vegetables, whole grains, low‐fat dairy, and nuts and legumes was related to a lower risk for AAA.

**Conclusions:**

Greater adherence to a Dietary Approaches To Stop Hypertension‐style dietary pattern was associated with lower risk for AAA. Higher consumption of fruits, vegetables, whole grains, low‐fat dairy as well as nuts and legumes may help to decrease the burden of AAAs.

**Clinical Perspective**

**What Is New?**

- The role of a healthy dietary pattern in the prevention of abdominal aortic aneurysms is largely unknown.

**What Are the Clinical Implications?**

- In a community‐based setting, individuals with high adherence to a Dietary Approaches To Stop Hypertension‐style dietary pattern had a 40% lower risk of hospitalization for abdominal aortic aneurysm than those with low adherence.
- Higher consumption of fruits, vegetables, whole grains, low‐fat dairy as well as nuts and legumes may help to decrease the burden of abdominal aortic aneurysms in US adults.

## Introduction

Abdominal aortic aneurysms (AAAs) develop in up to 8% of men and 6% of women over a lifetime.^1^ Although at most times they remain asymptomatic, rupture of an AAA carries high mortality.^2^ Aortic wall inflammation has been shown to possibly predict AAA expansion, rupture, and need for surgical repair.^2^, ^3^ However, given the considerable morbidity and mortality risk associated with AAA progression, understanding the role of risk factors and preventive measures is of great clinical importance. First and foremost, smoking followed by hypertension and hypercholesterolemia have been identified as modifiable risk factors for developing AAAs, although both latter ones have shown inconsistent findings.^1^, ^4^, ^5^, ^6^ The role of diet in the development of AAAs is largely unclear. Most evidence is confined to single food items or nutrients, and findings are inconsistent across studies.^4^, ^7^, ^8^, ^9^, ^10^ Information is lacking with regards to the association between adherence to a healthy eating pattern and AAA risk in the general population.

The Dietary Approaches to Stop Hypertension (DASH) diet is a healthy and balanced dietary pattern currently promoted by the National Heart, Lung, and Blood Institute of the National Institutes of Health, the United States Department of Agriculture, and the American Heart Association/American College of Cardiology as a healthy diet for all Americans.^11^, ^12^, ^13^, ^14^, ^15^ It is rich in fruits, vegetables, low‐fat dairy and includes mostly whole grains; lean meats, fish and poultry; nuts and beans. Adhering to a DASH dietary pattern has been demonstrated to effectively lower blood pressure levels^14^, ^16^ and, in observational studies, it has been shown to be associated with a reduced risk of cardiovascular events and kidney disease.^17^, ^18^, ^19^

The objective of this study was to assess if adherence to a DASH‐style dietary pattern is associated with an altered risk for AAA. We therefore examined the association between adherence to DASH‐style dietary pattern and its components with subsequent incidence of AAA in a large, US community‐based cohort of middle‐aged adults. We hypothesized that adherence to a DASH‐style dietary pattern would be associated with a lower risk for AAAs. Second, given previous data suggesting a close linkage between chronic inflammation and the development of AAA,^3^, ^20^ we further hypothesized that the relationship between a DASH‐style dietary pattern and AAA risk would differ by the presence of systemic inflammation reflected by C‐reactive protein (CRP) levels.

## Methods

The data, analytic methods, and study materials are made available to other researchers for purposes of reproducing the results or replicating the procedure. The data underlying our work can be obtained through 2 mechanisms. First, interested investigators can contact the ARIC Coordinating Center at the University of North Carolina—Chapel Hill. Details about the procedures for data request can be found online.^21^ Second, most ARIC data can be obtained from BioLINCC, a repository maintained by the National Heart, Lung, and Blood Institute. The BioLINCC website includes detailed information about the available data and the process to obtain such data.^22^

### Study Population

The ARIC (Atherosclerosis Risk in Communities) study is a community‐based prospective cohort study of 15 792 middle‐aged adults (aged 45–64 years at baseline) from 4 US communities (Washington County, Maryland; suburban Minneapolis, Minnesota; Jackson, Mississippi; and Forsyth County, North Carolina).^23^ ARIC investigators performed a baseline examination of participants in 1987–1989 (visit 1), and followed them by annual telephone interview and up to 4 re‐examinations in 1990–1992 (visit 2), 1993–1995 (visit 3), 1996–1998 (visit 4), and 2011–2013 (visit 5). For this analysis, only white and black adults were included; blacks from the Minneapolis and Washington County field centers were excluded because of small numbers (n=103). Furthermore, we excluded individuals with previous surgery for AAA (reporting prior AAA surgery or aortic angioplasty) and those whose follow‐up AAA status was uncertain at baseline (n=41).^1^, ^20^ Participants with incomplete dietary information or with implausible total energy intake (<600 or >4200 kcal per day for men, <500 or >3600 kcal per day for women) were also excluded from the analysis (n=359). Finally, individuals with missing dietary data or covariate information were not part of this analysis (n=1793). Our final study sample consisted of 13 496 participants who were followed through December 31, 2011 (Figure).

**Figure 1:**
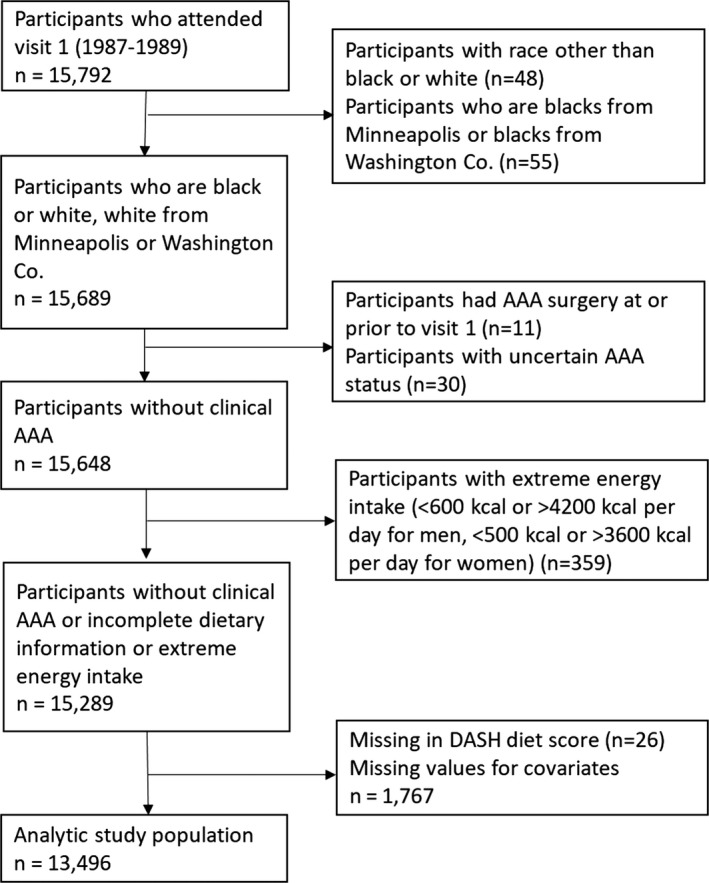
Flow diagram for selection of the analytic study population. AAA indicates abdominal aortic aneurysm; DASH, Dietary Approaches to Stop Hypertension.

All participating institutions received institutional review board approval for the study and all participants provided written informed consent.

### Assessment of Dietary Intake

The ARIC study assessed dietary intake using a 66‐item food frequency questionnaire (FFQ) adapted from the 61‐item FFQ developed by Willett et al^24^ which has been shown to be reliable.^25^ The FFQ was administered in person to all subjects by a trained interviewer following a standard protocol at visit 1 (1987–1989) and visit 3 (1993–1995). The cumulative average diet incorporating the 2 dietary assessments, when available before AAA occurrence, was used in the analysis, as this improves the estimation of usual dietary intake relative to a single assessment.^26^

### Assessment of DASH Diet Score

Two previously developed dietary scoring indices based on food items or nutrient targets were applied to assess adherence to a DASH‐style diet.^19^, ^27^, ^28^, ^29^ The DASH diet score based predominantly on food items considered intake of: (1) fruits, (2) vegetables, (3) nuts and legumes, (4) low‐fat dairy products, (5) whole grains, (6) sodium, (7) sweetened beverages, and (8) red and processed meats.^19^, ^30^ For fruits, vegetables, nuts and legumes, low‐fat dairy, and whole grains, participants in the highest quintile received a score of 5, those in the second highest quintile received a score of 4, and so on. For sodium, sweetened beverages, and red and processed meats, scoring was reversed, ie, participants in the highest quintile received a minimum score of 1 whereas participants in the lowest quintile received a maximum score of 5. The scores for each component were summed and the overall score ranged from 8 (no adherence) to 40 (perfect adherence).^19^

In sensitivity analyses, we applied the DASH diet score based on nutrient targets. This score consisted of 9 components, ie, low intake of (1) saturated fat, (2) total fat, (3) cholesterol, and (4) sodium and high intake of (5) protein, (6) fiber, (7) magnesium, (8) calcium, and (9) potassium.^27^, ^28^, ^29^ Individuals who met the DASH target for a nutrient received a score of 1 while those who achieved the intermediate target for a nutrient received a score of 0.5, and those meeting neither goal received 0 points. These DASH nutrient component scores were summed and ranged from 0 to 9 with 9 points representing optimal accordance with a DASH dietary pattern.^27^, ^28^, ^29^

Both scoring systems were analyzed using quintiles with a higher quintile reflecting higher adherence to a DASH‐style diet. We also analyzed quintiles of individual food and nutrient components (including sodium/potassium ratio) of the DASH diet scores. Study participants did not receive dietary counseling, and the DASH‐style diet results were not published at the time of ARIC study visits 1 and 3.^14^

### Assessment of Abdominal Aortic Aneurysms

The primary end point for this study was incident AAA after baseline (ARIC visit 1, 1987–1989). Incident clinical AAAs were identified by searching hospitalization and death records as well as Medicare data as previously outlined.^1^, ^20^ In annual telephone calls with ARIC participants, interviewers asked about any interim hospitalizations and identified deaths, and these hospitalization and death records were sought. Surveillance was conducted to identify additional hospitalizations or deaths and linked participant identifiers with Medicare data from the Centers for Medicare and Medicaid Services for 1991–2011, to find any missing hospital or outpatient events for those aged >65 years. Clinical AAAs were identified as those with a hospital discharge diagnosis, or 2 Medicare outpatient claims that occurred at least 1 week apart, with *International Classification of Diseases, Ninth Revision, Clinical Modification (ICD‐9‐CM)* codes of 441.3 or 441.4, or procedure codes of 38.44 or 39.71, or the following cause of death codes: *ICD‐9* 441.3 or 441.4 or *ICD‐10* code I71.3 or I71.4.^1^ Although labeled “clinical AAAs,” these diagnoses included both symptomatic and asymptomatic AAAs that were medically documented. Thoracic, thoracoabdominal, or unspecified aortic aneurysms were treated as non‐events.

### Covariates

Covariates were assessed at baseline via standardized protocols or questionnaires.^23^, ^31^ Information on smoking habits was obtained by interview and participants were classified as current, former, or never smokers. Pack‐years of smoking were calculated as the average number of cigarettes smoked per day multiplied by the years of smoking divided by 20 (the number of cigarettes in a standard pack).^1^ Data on education and intake of antihypertensive or lipid lowering medication were derived from standardized questionnaires.^23^ Sports‐related physical activity and leisure‐related physical activity were assessed with the use of Baecke's questionnaire and scoring systems.^32^ Hypertension was coded positive in participants with systolic blood pressure ≥140 mm Hg or diastolic blood pressure ≥90 mm Hg or use of antihypertensive medication. Abdominal obesity was defined as waist‐to‐hip ratio >0.85 for females and >0.90 for males. ARIC participants underwent venipuncture at each examination.^23^ Diabetes mellitus status was coded positive in participants with self‐reported physician diagnosis of diabetes mellitus, fasting blood glucose ≥126 mg/dL, non‐fasting blood glucose ≥200 mg/dL, or use of diabetes mellitus medication. Hypercholesterolemia was coded positive if total blood cholesterol level ≥240 mg/dL or if the participant was taking cholesterol‐lowering drugs. Cardiovascular disease was defined as self‐reported history of myocardial infarction, adjudicated myocardial infarction on ECG, coronary bypass, or angioplasty. At ARIC visit 2, CRP was measured in serum using a latex‐particle enhanced immunoturbidimetric assay kit (Roche Diagnostics, Indianapolis, IN) and read on the Roche Modular P800 Chemistry analyzer (Roche Diagnostics).^33^

### Statistical Analysis

Means and proportions were used to describe baseline characteristics (ARIC visit 1, 1987–1989) according to quintiles of the food‐based DASH‐style diet score. To assess the crude (unadjusted) association of the DASH‐style diet score with AAAs, we calculated incidence rates (IR) of incident AAA per 1000 person‐years as the number of diagnosed cases of AAA occurring during the entire follow‐up period divided by person‐years of follow‐up according to quintile of the DASH‐style diet score. Time to event was defined as time from the baseline examination to the date of AAA identification, death, lost to follow‐up, or December 31, 2011. Cox proportional hazards regression models were used to account for potential confounding on the association between the DASH‐style diet score and incident AAAs. An initial model adjusted for sex, total energy intake, race‐center, and age by the use of restricted cubic splines with knots at 45, 51, 57, and 63 years of age representing the 5th, 35th, 65th, and 95th percentiles (minimally adjusted model; model 1). A second model adjusted for all of the baseline covariates in model 1 plus alcohol intake quintiles (sex specific quintiles of g/week), education (less than high school, high school or equivalent, college or above), household income (<$25 000; $25 000–$49 999; ≥$50 000), smoking status (current smoker, former smoker with ≥20 pack‐years, former smoker with <20 pack‐years, never smoker), sport‐related physical activity index, leisure‐time physical activity index, body mass index category (<25, 25 to <30, ≥30 kg/m^2^), abdominal obesity (waist‐to‐hip ratio >0.85 for females and >0.90 for males), hypertension (yes/no), diabetes mellitus (yes/no), hypercholesterolemia (yes/no), and cardiovascular disease (yes/no) (fully adjusted model; model 2).^7^ We tested for trend across quintiles of the DASH‐style diet score using the median value within each quintile. Besides examining the overall dietary pattern, we further examined the relationship between individual food and nutrient components of the DASH style dietary scores and risk for AAA, including each component separately in the fully adjusted model. Additional analyses were undertaken by including time‐varying cases of hypertension into the modeling with further stratification by CRP levels (above versus below or equal to 3 mg/L). Finally, we examined effect modification by sex, race, smoking, and obesity using stratified analyses and tests of interaction with each of the potential modifiers. Stata version 14.2 was used for all statistical analyses (StataCorp LP, College Station, TX).

## Results

Individuals with a DASH diet score in the highest quintile were older, more likely to be female, to be white, to have a higher education and a higher household income, to have never smoked, and to have lower CRP levels (Table 1).

**Table 1:** Baseline Characteristics According to Quintiles of DASH Diet Score, ARIC, 1987–1989

Characteristic	Quintiles of Food‐Based DASH Diet Score^a^: Median (Minimum–Maximum), n					*P* Value for Trend^b^
	Quintile 1: 17 (8–19), n=2670	Quintile 2: 22 (20–23), n=3425	Quintile 3: 25 (24–25), n=1979	Quintile 4: 27 (26–28), n=2686	Quintile 5: 31 (29–38), n=2736	
Age, y	53.4 (5.7)	53.8 (5.7)	54.1 (5.7)	54.5 (5.7)	55.2 (5.7)	<0.001
Female, %	1059 (39.7%)	1642 (47.9%)	1120 (56.6%)	1656 (61.7%)	1934 (70.7%)	<0.001
Black, %	987 (37.0%)	932 (27.2%)	431 (21.8%)	500 (18.6%)	407 (14.9%)	<0.001
Smoking status, %						<0.001
Current smoker	1049 (39.3%)	1040 (30.4%)	448 (22.6%)	548 (20.4%)	426 (15.6%)	
Former smoker, ≥20 pack‐years	389 (14.6%)	539 (15.7%)	278 (14.0%)	415 (15.5%)	382 (14.0%)	
Former smoker, <20 pack‐years	373 (14.0%)	526 (15.4%)	348 (17.6%)	505 (18.8%)	581 (21.2%)	
Never smoker	859 (32.2%)	1320 (38.5%)	905 (45.7%)	1218 (45.3%)	1347 (49.2%)	
Education, %						<0.001
Less than high school	960 (36.0%)	857 (25.0%)	386 (19.5%)	454 (16.9%)	392 (14.3%)	
High school or equivalent	1102 (41.3%)	1444 (42.2%)	852 (43.1%)	1120 (41.7%)	1046 (38.2%)	
College or above	608 (22.8%)	1124 (32.8%)	741 (37.4%)	1112 (41.4%)	1298 (47.4%)	
Annual househould income, %						<0.001
<$24 999	1260 (47.2%)	1326 (38.7%)	667 (33.7%)	883 (32.9%)	841 (30.7%)	
$25 000 to $49 999	995 (37.3%)	1270 (37.1%)	775 (39.2%)	1000 (37.2%)	1056 (38.6%)	
>$50 000	415 (15.5%)	829 (24.2%)	537 (27.1%)	803 (29.9%)	839 (30.7%)	
Diabetes mellitus,^c^ %	220 (8.2%)	365 (10.7%)	254 (12.8%)	359 (13.4%)	365 (13.3%)	<0.001
Prevalent CVD, %	191 (7.2%)	312 (9.1%)	159 (8.0%)	244 (9.1%)	278 (10.2%)	0.001
Hypertension,^d^ %	1038 (38.9%)	1328 (38.8%)	757 (38.3%)	1061 (39.5%)	1068 (39.0%)	0.719
Hypercholesterolemia,^e^ %	634 (23.7%)	888 (25.9%)	557 (28.1%)	735 (27.4%)	775 (28.3%)	<0.001
BMI category, %						<0.001
BMI <25 kg/m^2^	877 (32.8%)	1085 (31.7%)	600 (30.3%)	887 (33.0%)	1062 (38.8%)	
BMI 25 to <30 kg/m^2^	1059 (39.7%)	1388 (40.5%)	840 (42.4%)	1047 (39.0%)	1025 (37.5%)	
BMI ≥30 kg/m^2^	734 (27.5%)	952 (27.8%)	539 (27.2%)	752 (28.0%)	649 (23.7%)	
Abdominal obesity,^f^ %	2198 (82.3%)	2760 (80.6%)	1538 (77.7%)	2052 (76.4%)	1997 (73.0%)	<0.001
Leisure‐time physical activity index	2.1 (0.5)	2.3 (0.6)	2.4 (0.5)	2.5 (0.6)	2.6 (0.6)	<0.001
Sport‐related physical activity index	2.2 (0.7)	2.4 (0.8)	2.4 (0.8)	2.5 (0.8)	2.7 (0.8)	<0.001
Total energy intake, kcal/d	1760.9 (638.2)	1656.0 (648.7)	1608.3 (611.1)	1566.1 (587.3)	1536.4 (518.4)	<0.001
C‐reactive protein,^g^ mg/L	4.7 (7.9)	4.5 (6.3)	4.6 (8.6)	4.2 (6.9)	3.9 (6.0)	<0.001

Data reported are mean (standard deviation) or n (%). ARIC indicates Atherosclerosis Risk in Communities Study; BMI, body mass index; CVD, cardiovascular disease; DASH, Dietary Approaches to Stop Hypertension.

a Food consumption (DASH diet score and individual nutrients) was estimated using cumulative average intake. For those who developed AAA or were censored from the analysis before visit 3, food frequency questionnaire data from visit 1 were used. Otherwise, for those who developed AAA or were censored from the analysis after study visit 3, the average of food frequency questionnaire data from visits 1 and 3 were used.

b Cochran‐Armitage trend tests for categorical variables and linear regression for continuous variables were used to test for trend in baseline characteristics across quintiles of DASH diet scores.

c Diabetes mellitus status was defined as self‐reported physician diagnosis of diabetes mellitus, fasting blood glucose ≥126 mg/dL, non‐fasting blood glucose ≥200 mg/dL, or current use of diabetes mellitus medication.

d Hypertension was defined as systolic blood pressure ≥140 mm Hg, diastolic blood pressure ≥90 mm Hg, or current use of anti‐hypertensive medication.

e Hypercholesterolemia was defined as total blood cholesterol level ≥240 mg/dL or current use of lipid‐lowering medication.

f Abdominal obesity was defined as waist‐to‐hip ratio >0.85 for females and >0.90 for males.

g C‐reactive protein (CRP) was measured at ARIC visit 2 (1990–1992). Among 13 080 participants attending visit 2, 12 227 participants had measurements of CRP.

A total of 517 cases of incident hospitalized AAA cases were observed during a median follow‐up of 23 years among 13 496 participants. Across quintiles of increasing DASH dietary scores based on food items, individuals with greatest self‐reported adherence had a lower risk for incident AAA hospitalizations (Table 2). Participants in the highest quintile had a 40% lower risk of developing incident AAA than those with the lowest score quintile (HR_Q5 vs Q1_: 0.60; 95% CI: 0.44, 0.83; *P*_trend_=0.002) after fully adjusting for confounding variables (model 2). These main results did not change substantially when we excluded current smokers at baseline from our analyses (Table S1) or undertook stratified analyses by sex (Table S2). Furthermore, our results also held true for sensitivity analyses using a DASH diet score based on nutrient targets (HR_Q5 vs Q1_: 0.61; 95% CI: 0.45, 0.84; *P*_trend_=0.006) (Table S3). When we investigated the interactions of adherence to a DASH‐style dietary pattern with AAA risk, we did not observe any significant interaction by sex (*P*_interaction by sex_=0.32), race (*P*_interaction by race_=0.38), smoking (*P*_interaction by smoking_=0.79), or obesity (*P*_interaction by obesity_=0.16).

**Table 2:** Association of Quintiles of Food‐Based DASH Diet Score With Incident AAA^a^

	Quintiles of Food‐Based DASH Diet Score^b^					*P* Value for Trend^c^
	Quintile 1: n=2670	Quintile 2: n=3425	Quintile 3: n=1979	Quintile 4: n=2686	Quintile 5: n=2736	
Median Score (range)	17.0 (8.0–19.0)	22.0 (20.0–23.0)	25.0 (24.0–25.0)	27.0 (26.0–28.0)	31.0 (29.0–38.0)	
Events	144	151	61	94	67	
Person‐years	50 560	67 163	39 767	54 502	55 991	
IR (95% CI)^d^	2.85 (2.42–3.35)	2.25 (1.92–2.64)	1.53 (1.19–1.97)	1.72 (1.41–2.11)	1.20 (0.94–1.52)	
Model 1 HR (95% CI)^e^	1 (ref)	0.76 (0.61–0.96)	0.53 (0.39–0.72)	0.59 (0.45–0.77)	0.41 (0.31–0.56)	<0.001
Model 2 HR (95% CI)^f^	1 (ref)	0.87 (0.69–1.10)	0.69 (0.51–0.94)	0.79 (0.60–1.05)	0.60 (0.44–0.83)	0.002

AAA indicates abdominal aortic aneurysm; ARIC, Atherosclerosis Risk in Communities Study; CI, confidence interval; DASH, Dietary Approaches to Stop Hypertension; HR, hazard ratio; IR, incidence rate.

a Incident AAA cases were ascertained from baseline (1987–1989) through December 31, 2011.

b Food consumption (DASH diet score and individual components) was estimated using cumulative average intake. For those who developed AAA or were censored from the analysis before visit 3, food frequency questionnaire data from visit 1 were used. Otherwise, for those who developed AAA or were censored from the analysis after study visit 3, the average of food frequency questionnaire data from visits 1 and 3 were used.

c Trend across quintiles was tested using the median value for the DASH diet score within each quintile.

d IR (incidence rate) expressed as the number of AAA cases per 1000 person‐years with no adjustment for covariates.

e Model 1: adjusted for sex, total energy intake, race‐center, and age by the use of restricted cubic splines (with knots at 45, 51, 57, and 63 years of age, representing the 5th, 35th, 65th, and 95th percentiles).

f Model 2: adjusted for variables in model 1+alcohol intake quintiles (sex specific quintiles of g/week), education (less than high school, high school or equivalent, college or above), household income (<$25 000; $25 000–$49 999; ≥$50 000), smoking status (current smoker, former smoker with ≥20 pack‐years, former smoker with <20 pack‐years, never smoker), sport‐related physical activity index, leisure‐time physical activity index, body mass index category (<25, 25 to <30, ≥30 kg/m^2^), abdominal obesity (waist‐to‐hip ratio >0.85 for females and >0.90 for males), hypertension (yes/no), diabetes mellitus (yes/no), hypercholesterolemia (yes/no), and cardiovascular disease (yes/no).

In detailed analyses of food‐item components of a DASH‐style dietary pattern (Table 3), individuals with the highest levels of dietary intake of fruits, vegetables, whole grains, and low‐fat dairy products had a lower risk of incident AAA (fruits HR_Q5 vs Q1_: 0.70; 95% CI: 0.52, 0.95, *P*_trend_=0.013; vegetables HR_Q5 vs Q1_: 0.60; 95% CI: 0.44, 0.80, *P*_trend_<0.001; whole grains HR_Q5 vs Q1_: 0.67; 95% CI: 0.51, 0.89, *P*_trend_=0.027; low‐fat dairy HR_Q5 vs Q1_: 0.48; 95% CI: 0.36, 0.63, *P*_trend_<0.001). In addition, higher nuts and legumes consumption was associated with reduced AAA risk (HR_Q5 vs Q1_: 0.55; 95% CI: 0.41, 0.72, *P*_trend_<0.001). Higher sodium intake was significantly associated with a decreased risk for incident AAAs (HR_Q5 vs Q1_: 0.64; 95% CI: 0.44, 0.93, *P*_trend_=0.006), whereas a higher sodium‐potassium ratio was not associated with AAA risk (HR_Q5 vs Q1_: 0.86; 95% CI: 0.66, 1.12, *P*_trend_=0.409). Dietary intake of red and processed meat products as well as sweetened beverages were not significantly associated with AAA risk in the fully adjusted model. In detailed analyses of nutrient‐item components of a DASH‐style dietary pattern, lower saturated fat, higher protein, and higher fiber intake were associated with a lower risk for incident AAAs (Table S4).

**Table 3:** Association of Individual Components of the Food‐Based DASH Diet Score and Risk for AAA^a^

Component	Quintiles of DASH Food Components^b^					*P* Value for Trend^c^
	Quintile 1	Quintile 2	Quintile 3	Quintile 4	Quintile 5	
Red and processed meat						
Median (range), svg/d	0.3 (0.0–0.5)	0.6 (0.5–0.8)	0.9 (0.8–1.1)	1.3 (1.1–1.6)	2.0 (1.6–13.6)	
Events	103	88	67	113	146	
Person‐years	55 341	55 966	54 641	52 190	49 844	
IR (95% CI)^d^	1.86 (1.53–2.26)	1.57 (1.28–1.94)	1.23 (0.97–1.56)	2.17 (1.80–2.60)	2.93 (2.49–3.44)	
Model 1 HR (95% CI)^e^	1 (ref)	0.86 (0.65–1.15)	0.60 (0.44–0.83)	1.01 (0.76–1.35)	1.33 (0.98–1.80)	0.039
Model 2 HR (95% CI)^f^	1 (ref)	0.81 (0.61–1.08)	0.54 (0.40–0.75)	0.88 (0.66–1.18)	1.04 (0.77–1.42)	0.563
Sweetened beverages						
Median (range), svg/d	0.0 (0.0–0.0)	0.1 (0.0–0.1)	0.2 (0.1–0.4)	0.6 (0.5–1.0)	1.4 (1.0–9.5)	
Events	152	53	128	65	119	
Person‐years	57 561	52 659	56 847	50 182	50 733	
IR (95% CI)^d^	2.64 (2.25–3.10)	1.01 (0.77–1.32)	2.25 (1.89–2.68)	1.30 (1.02–1.65)	2.35 (1.96–2.81)	
Model 1 HR (95% CI)^e^	1 (ref)	0.33 (0.24–0.45)	0.69 (0.54–0.87)	0.40 (0.30–0.54)	0.82 (0.63–1.08)	0.127
Model 2 HR (95% CI)^f^	1 (ref)	0.37 (0.27–0.51)	0.76 (0.59–0.97)	0.43 (0.32–0.58)	0.80 (0.60–1.05)	0.071
Fruits						
Median (range), svg/d	0.6 (0.0–0.9)	1.3 (1.0–1.6)	1.9 (1.6–2.2)	2.6 (2.2–3.1)	3.9 (3.1–23.6)	
Events	146	106	107	79	79	
Person‐years	50 049	53 534	55 904	54 700	53 796	
IR (95% CI)^d^	2.92 (2.48–3.43)	1.98 (1.64–2.40)	1.91 (1.58–2.31)	1.44 (1.16–1.80)	1.47 (1.18–1.83)	
Model 1 HR (95% CI)^e^	1 (ref)	0.63 (0.49–0.81)	0.60 (0.47–0.78)	0.47 (0.35–0.62)	0.47 (0.35–0.62)	<0.001
Model 2 HR (95% CI)^f^	1 (ref)	0.76 (0.59–0.98)	0.80 (0.62–1.04)	0.68 (0.51–0.91)	0.70 (0.52–0.95)	0.013
Vegetables						
Median (range), svg/d	0.4 (0.0–0.6)	0.8 (0.6–0.9)	1.1 (0.9–1.3)	1.5 (1.3–1.8)	2.3 (1.8–18.1)	
Events	141	122	92	91	71	
Person‐years	52 883	53 115	54 245	54 117	53 623	
IR (95% CI)^d^	2.67 (2.26–3.14)	2.30 (1.92–2.74)	1.70 (1.38–2.08)	1.68 (1.37–2.07)	1.32 (1.05–1.67)	
Model 1 HR (95% CI)^e^	1 (ref)	0.85 (0.66–1.08)	0.63 (0.48–0.82)	0.62 (0.48–0.82)	0.48 (0.36–0.65)	<0.001
Model 2 HR (95% CI)^f^	1 (ref)	0.98 (0.77–1.25)	0.73 (0.56–0.96)	0.72 (0.55–0.95)	0.60 (0.44–0.80)	<0.001
Whole grains						
Median (range), svg/d	0.1 (0.0–0.2)	0.4 (0.3–0.6)	0.7 (0.6–0.9)	1.1 (0.9–1.5)	2.2 (1.6–8.6)	
Events	149	115	51	121	81	
Person‐years	53 932	55 711	50 401	52 906	55 032	
IR (95% CI)^d^	2.76 (2.35–3.24)	2.06 (1.72–2.48)	1.01 (0.77–1.33)	2.29 (1.91–2.73)	1.47 (1.18–1.83)	
Model 1 HR (95% CI)^e^	1 (ref)	0.76 (0.60–0.97)	0.38 (0.28–0.53)	0.83 (0.65–1.05)	0.47 (0.36–0.62)	<0.001
Model 2 HR (95% CI)^f^	1 (ref)	0.92 (0.72–1.17)	0.48 (0.35–0.66)	1.00 (0.79–1.28)	0.67 (0.51–0.89)	0.027
Low‐fat dairy						
Median (range), svg/d	0.0 (0.0–0.1)	0.2 (0.1–0.4)	0.6 (0.5–0.9)	1.0 (0.9–1.4)	2.1 (1.4–10.8)	
Events	181	85	25	135	91	
Person‐years	52 848	51 281	53 785	54 743	55 326	
IR (95% CI)^d^	3.42 (2.96–3.96)	1.66 (1.34–2.05)	0.46 (0.31–0.69)	2.47 (2.08–2.92)	1.64 (1.34–2.02)	
Model 1 HR (95% CI)^e^	1 (ref)	0.43 (0.33–0.55)	0.11 (0.07–0.16)	0.55 (0.44–0.70)	0.37 (0.28–0.48)	<0.001
Model 2 HR (95% CI)^f^	1 (ref)	0.48 (0.37–0.63)	0.13 (0.08–0.20)	0.71 (0.56–0.90)	0.48 (0.36–0.63)	<0.001
Nuts and legumes						
Median (range), svg/d	0.3 (0.0–0.4)	0.6 (0.4–0.7)	0.8 (0.7–0.9)	1.1 (0.9–1.3)	1.7 (1.3–10.6)	
Events	130	90	86	110	101	
Person‐years	52 229	54 578	53 936	54 070	53 170	
IR (95% CI)^d^	2.49 (2.10–2.96)	1.65 (1.34–2.03)	1.59 (1.29–1.97)	2.03 (1.69–2.45)	1.90 (1.56–2.31)	
Model 1 HR (95% CI)^e^	1 (ref)	0.59 (0.45–0.78)	0.54 (0.41–0.71)	0.63 (0.48–0.82)	0.49 (0.37–0.65)	<0.001
Model 2 HR (95% CI)^f^	1 (ref)	0.63 (0.48–0.83)	0.60 (0.45–0.79)	0.69 (0.53–0.90)	0.55 (0.41–0.72)	<0.001
Sodium						
Median (range), mg/d	848 (260–1019)	1153 (1020–1285)	1412 (1285–1550)	1704 (1550–1907)	2211 (1907–5030)	
Events	103	103	86	94	131	
Person‐years	52 138	54 684	54 793	53 846	52 522	
IR (95% CI)^d^	1.98 (1.63–2.40)	1.88 (1.55–2.28)	1.57 (1.27–1.94)	1.75 (1.43–2.14)	2.49 (2.10–2.96)	
Model 1 HR (95% CI)^e^	1 (ref)	0.80 (0.60–1.05)	0.59 (0.43–0.79)	0.57 (0.41–0.78)	0.65 (0.45–0.94)	0.004
Model 2 HR (95% CI)^f^	1 (ref)	0.78 (0.59–1.03)	0.59 (0.44–0.81)	0.59 (0.43–0.82)	0.64 (0.44–0.93)	0.006
Sodium/potassium ratio^g^						
Median (range)	0.4 (0.1–0.5)	0.5 (0.5–0.5)	0.6 (0.5–0.6)	0.6 (0.6–0.7)	0.8 (0.7–2.0)	
Events	118	79	91	95	134	
Person‐years	53 802	54 383	54 506	54 143	51 148	
IR (95% CI)^d^	2.19 (1.83–2.63)	1.45 (1.17–1.81)	1.67 (1.36–2.05)	1.75 (1.43–2.15)	2.62 (2.21–3.10)	
Model 1 HR (95% CI)^e^	1 (ref)	0.60 (0.45–0.79)	0.64 (0.49–0.84)	0.64 (0.49–0.84)	0.94 (0.73–1.21)	0.992
Model 2 HR (95% CI)^f^	1 (ref)	0.61 (0.45–0.81)	0.66 (0.50–0.87)	0.63 (0.48–0.83)	0.86 (0.66–1.12)	0.409

AAA indicates abdominal aortic aneurysm; ARIC, Atherosclerosis Risk in Communities Study; CI, confidence interval; DASH, Dietary Approaches to Stop Hypertension; HR, hazard ratio; IR, incidence rate; svg/d, servings/day.

a Incident AAA cases were ascertained from baseline (1987–1989) through December 31, 2011.

b Food consumption was estimated using cumulative average intake. For those who developed AAA or were censored from the analysis before visit 3, food frequency questionnaire data from visit 1 were used. Otherwise, for those who developed AAA or were censored from the analysis after study visit 3, the average of food frequency questionnaire data from visits 1 and 3 were used.

c Trend across quintiles was tested using the median value for the component of the DASH diet score within each quintile.

d IR (incidence rate) expressed as the number of AAA cases per 1000 person‐years with no adjustment for covariates.

e Model 1: adjusted for sex, total energy intake, race‐center, and age by the use of restricted cubic splines (with knots at 45, 51, 57, and 63 years of age, representing the 5th, 35th, 65th, and 95th percentiles).

f Model 2: adjusted for variables in model 1+alcohol intake quintiles (sex specific quintiles of g/week), education (less than high school, high school or equivalent, college or above), household income (<$25 000; $25 000–$49 999; ≥$50 000), smoking status (current smoker, former smoker with ≥20 pack‐years, former smoker with <20 pack‐years, never smoker), sport‐related physical activity index, leisure‐time physical activity index, body mass index category (<25, 25 to <30, ≥30 kg/m^2^), abdominal obesity (waist‐to‐hip ratio >0.85 for females and >0.90 for males), hypertension (yes/no), diabetes mellitus (yes/no), hypercholesterolemia (yes/no), and cardiovascular disease (yes/no).

g Sodium/potassium ratio was defined as (Na mg/1000 kcal)/(K mg/1000 kcal).

To assess for partial mediation of the association between the DASH style dietary pattern and incident AAA risk, we additionally adjusted for hypertension modeled as a time‐varying variable. Our results changed only marginally (Table 4). After further stratifying our time‐varying results by CRP level, we found that participants with CRP levels above 3 mg/L tended to have a stronger inverse association between the food‐based DASH diet score and risk of incident AAA compared with people with CRP ≤3 mg/L (*P*_interaction_=0.130).

**Table 4:** Association of Quintiles of DASH Diet Score With Incident AAA^a^ After Adjusting for Time‐Varying Hypertension and Stratified by C‐Reactive Protein Levels

	Quintiles of DASH Diet Score^b^					*P* Value for Trend^c^	*P* for Interaction
	Quintile 1: n=2870	Quintile 2: n=3642	Quintile 3: n=2085	Quintile 4: n=2834	Quintile 5: n=2891		
Overall^d^	1 (ref)	0.80 (0.62–1.02)	0.64 (0.46–0.88)	0.72 (0.54–0.96)	0.53 (0.38–0.74)	<0.001	
CRP ≤3 mg/L^d^	1 (ref)	0.84 (0.58–1.23)	0.72 (0.45–1.14)	0.92 (0.61–1.37)	0.58 (0.36–0.94)	0.091	
CRP >3 mg/L^d^	1 (ref)	0.66 (0.46–0.96)	0.41 (0.24–0.70)	0.44 (0.27–0.71)	0.45 (0.27–0.75)	<0.001	0.130

AAA indicates abdominal aortic aneurysm; ARIC, Atherosclerosis Risk in Communities Study; CI, confidence interval; CRP, C‐reactive protein; DASH, Dietary Approaches to Stop Hypertension; HR, hazard ratio; IR, incidence rate.

a Incident AAA cases were ascertained from ARIC visit 2 (1990–1992) through December 31, 2011.

b Food consumption was estimated using cumulative average intake. For those who developed AAA or were censored from the analysis before visit 3, food frequency questionnaire data from visit 1 were used. Otherwise, for those who developed AAA or were censored from the analysis after study visit 3, the average of food frequency questionnaire data from visits 1 and 3 were used.

c Trend across quintiles was tested using the median value for the component of the DASH diet score within each quintile.

d Adjusted for sex, total energy intake, race‐center, and age by the use of restricted cubic splines (with knots at 45, 51, 57, and 63 years of age, representing the 5th, 35th, 65th, and 95th percentiles), alcohol intake quintiles (sex specific quintiles of g/week), education (less than high school, high school or equivalent, college or above), household income (<$25 000; $25 000–$49 999; ≥$50 000), smoking status (current smoker, former smoker with ≥20 pack‐years, former smoker with <20 pack‐years, never smoker), sport‐related physical activity index, leisure‐time physical activity index, body mass index category (<25, 25 to <30, ≥30 kg/m^2^), abdominal obesity (waist‐to‐hip ratio >0.85 for females and >0.90 for males), hypercholesterolemia (yes/no), cardiovascular disease (yes/no), and time‐varying hypertension.

## Discussion

In a large, community‐based cohort of middle‐aged adults, greater self‐reported adherence to a DASH‐style dietary pattern was associated with a significantly lower risk of AAA hospitalization.

The majority of the prospective epidemiological evidence for a dietary association with incident AAAs stems from studies of dietary components involving individuals from Sweden and populations in the United States.^4^, ^7^, ^8^, ^9^ Whereas in previous publications fruits as well as moderate alcohol consumption were associated with a lower risk of AAAs, vegetables did not show a consistent relationship, leaving the question open if and how a healthy dietary pattern might affect incident AAAs.^7^, ^8^, ^9^, ^10^ Current evidence suggests that the development of AAAs is driven by different pathophysiologic mechanisms that seem to be associated with tissue degradation and inflammation.^2^, ^3^ Experimental basic research studies in mice show that angiotensin II infusions which significantly raise blood pressure levels can induce AAAs by elastin fiber disruption associated with macrophage infiltration, medial rupture and thrombosis, followed by luminal expansion with adventitial and tissue remodeling.^34^, ^35^, ^36^ When additionally fed a high saturated fat diet, mice infused with angiotensin II experience a considerably higher risk for AAAs.^37^

Our results for a heart healthy dietary pattern known to lower blood pressure extend previous reports on single food items suggesting that adherence to a healthy, DASH‐style eating plan can significantly decrease AAA risk. In our community‐based study population, a diet rich in vegetables, whole grains, low‐fat dairy products, and nuts and legumes shows promising results for reducing the risk of incident AAAs. On a nutrient basis, higher saturated fat intake was related to greater risk for AAA which is in line with previous experimental data,^34^, ^37^ whereas higher dietary intake of protein and fiber consumption was associated with lower risk of AAA. On the other hand, sodium consumption, but not sodium‐to‐potassium ratio, showed a significant association with AAA risk. However, when we separately examined questions on sodium or salt intake specifically added to foods, no significant relationship with AAA risk was detected (data not shown). Given the methodological difficulties of observational studies to assess the absolute amounts of sodium consumed, the relationship between sodium consumption and AAA risk warrants further research.^38^

Hypertension was not a strong mediator of the observed inverse association between adherence to a DASH‐style dietary pattern and incident AAA. This underscores, on the one hand, the controversial role of hypertension in the expansion of the AAAs, while it, on the other hand, suggests the presence of other explanatory factors.^6^, ^39^ In fact, participants in a pro‐inflammatory state, reflected by CRP levels greater than 3 mg/L, tended to show a slightly stronger association between the DASH‐style dietary pattern and risk of AAA. Our epidemiological findings reinforce the basic science notion that alterations of shear stress by blood pressure elevations together with aortic wall layer break down stimulated by a pro‐inflammatory environment appear to play a key role in the pathophysiology of AAAs.

These results have both clinical and scientific implications. Rather than individual food items, a DASH‐style eating plan, which includes a variety of healthy food choices, may protect against incident AAAs. Second, this study strengthens the concept of the beneficial immuno‐modulating effects of a healthy dietary pattern on inflammatory processes.^40^, ^41^ Thus, our results, in line with current dietary recommendations on the prevention of other cardiovascular disease, provide evidence for future AAA prevention through early lifestyle intervention, based on not‐smoking and following a healthy dietary pattern.^12^

The strengths of our study include a large diverse, community‐based, prospective cohort with long follow‐up, structured assessment of dietary intake, assessment of and adjustment for relevant covariates, and adjudicated outcome events. Nonetheless, there are several limitations. Our exposure, food intake, was assessed in the late 1980's and mid 1990's; thus, changing dietary habits and food supply over time may not have been adequately captured by self‐reported FFQs. Furthermore, although the nutritional data derived from these FFQs have been shown to be reliable, assessing true dietary adherence to a DASH dietary pattern over time was not possible in our study setting.^25^ It is also likely that self‐reported adherence to DASH dietary pattern may constitute a marker for other healthy behaviours which increases the risk for residual confounding. Moreover, the use of self‐reported dietary information is affected by some degree of misclassification. Absolute amounts of consumed nutrients (in particular sodium) were likely to be underestimated because of several methodological issues including the limited number of items on the questionnaire and lack of information on food brands and snack foods.^38^, ^42^ Additionally, salt and sodium‐containing condiments were not queried. On the outcome side, no screening ultrasound for AAA was done at baseline, nor was there a clinical exam which is similar to prior analyses.^7^, ^10^ At baseline, participants were explicitly asked if they had had surgery involving the vascular system and we excluded the few participants who reported prior surgery on the aorta. Assessment of clinical AAAs after baseline was limited to hospital and death *ICD* codes. Although the AAA codes seem specific, we tried to enhance the validity of our AAA outcome by excluding aortic aneurysms coded to uncertain sites because they may not have been abdominal.^1^, ^20^ In this context, although the primary end point of incident AAA appears to be largely dependent on healthcare records, and thus engagement with medical services, accounting for household income did not significantly change our results. Finally, as always with observational studies, residual and unmeasured confounding could be partly responsible for the results although we adjusted our analyses for a wide range of confounding factors. Excluding current smokers at baseline did not significantly change our main results.

In conclusion, greater self‐reported adherence to a DASH dietary pattern was found to be associated with lower risk of incident AAA.

## Sources of Funding

The Atherosclerosis Risk in Communities study has been funded by the National Heart, Lung, and Blood Institute, National Institutes of Health, Department of Health and Human Services, under contract numbers HHSN268201700001I, HHSN2682017000021, HHSN268201700003I, HHSN268201700004I, and HHSN268201700005I. Dr Selvin was supported by grants from the National Institutes of Health and National Institute of Diabetes and Digestive and Kidney Diseases (K24DK106414 and R01DK089174). Dr Tang was supported by a grant from the National Heart, Lung, and Blood Institute (R01 HL103695). Dr. Rebholz is supported by a mentored research scientist development award from the National Institute of Diabetes and Digestive and Kidney Diseases (K01 DK107782). Open access publishing was funded by the German Research Foundation (DFG) and the University of Würzburg.

## Disclosures

None.

## Supporting information


**Table S1.** Association of Quintiles of Food‐Based DASH Diet Score With Incident AAA Excluding Current Smokers*
**Table S2.** Association of Quintiles of Food‐Based DASH Diet Score With Incident AAA by Sex*
**Table S3.** Association of Quintiles of Nutrient Based DASH Diet Score With Incident AAA*
**Table S4.** Association of Individual Nutrients of the Nutrient‐Based DASH Diet Score With Risk for AAA*
