# Supplementary material for: Adherence to the Dietary Approaches to Stop Hypertension Dietary Pattern and Risk of Abdominal Aortic Aneurysm: Results From the ARIC Study
Source: J Am Heart Assoc. 2018 Nov 2;7(21):e009340. doi: 10.1161/JAHA.118.009340 (PMC6404186; doi:10.1161/JAHA.118.009340)
Supplement: Supplementary file 1 — Table S1. Association of Quintiles of Food‐Based DASH Diet Score With Incident AAA Excluding Current Smokers* Table S2. Association of Quintiles of Food‐Based DASH Diet Score With Incident AAA by Sex* Table S3. Association of Quintiles of Nutrient Based DASH Diet Score With Incident AAA* Table S4. Association of Individual Nutrients of the Nutrient‐Based DASH Diet Score With Risk for AAA* [file JAH3-7-e009340-s001.pdf]

SUPPLEMENTAL MATERIAL

Table S1. Association of Quintiles of Food-Based DASH Diet Score with incident AAA excluding current smokers.*

	Quintiles of Food-Based DASH Diet Score†					
	**Quintile 1:** n=1,621	**Quintile 2:** n=2,385	**Quintile 3:** n=1,531	**Quintile 4:** n=2,138	**Quintile 5:** n=2,310	**P-value for trend‡**
Median Score (Range)	17.0 (8.0-19.0)	22.0 (20.0-23.0)	25.0 (24.0-25.0)	27.0 (26.0-28.0)	31.0 (29.0-38.0)	
Events	55	69	33	55	45	
Person-years	32,537	48,795	31,372	44,345	47,945	
IR (95% CI)§	1.69 (1.30-2.20)	1.41 (1.12-1.79)	1.05 (0.75-1.48)	1.24 (0.95-1.62)	0.94 (0.70-1.26)	
Model 1 HR (95% CI)‖	1 (ref)	0.81 (0.57-1.16)	0.65 (0.42-1.01)	0.77 (0.52-1.13)	0.61 (0.41-0.92)	0.030
Model 2 HR (95% CI) #	1 (ref)	0.79 (0.55-1.14)	0.66 (0.42-1.03)	0.72 (0.48-1.07)	0.58 (0.37-0.89)	0.018

AAA, abdominal aortic aneurysm; ARIC, Atherosclerosis Risk in Communities Study; CI, confidence interval; DASH, Dietary Approaches to Stop Hypertension; HR, hazard ratio; IR, incidence rate.

* Incident AAA cases were ascertained from baseline (1987-1989) through December 31, 2011.

† Food consumption (DASH diet score and individual components) was estimated using cumulative average intake. For those who developed AAA or were censored from the analysis before visit 3, food frequency questionnaire data from visit 1 was used. Otherwise, for those who developed AAA or were censored from the analysis after study visit 3, the average of food frequency questionnaire data from visits 1 and 3 was used.

‡ Trend across quintiles was tested using the median value for the DASH diet score within each quintile.

§ IR (incidence rate) expressed as the number of AAA cases per 1,000 person-years with no adjustment for covariates.

‖ Model 1: Adjusted for sex, total energy intake, race-center, and age by the use of restricted cubic splines (with knots at 45, 51, 57, and 63 years of age, representing the 5th, 35th, 65th, and 95th percentiles).

Model 2: Adjusted for variables in model 1 + alcohol intake quintiles (sex specific quintiles of g/week), education (less than high school, high school or equivalent, college or above), household income (<$25,000; $25,000-$49,999; ≥$50,000), smoking status (former smoker with ≥20 pack-years, former smoker with <20 pack-years, never smoker), sport-related physical activity index, leisure-time physical activity index, body mass index category (<25, 25-<30, ≥30 kg/m^2), abdominal obesity (waist-to-hip ratio >0.85 for females and >0.90 for males), hypertension (yes/no), diabetes (yes/no), hypercholesterolemia (yes/no), and cardiovascular disease (yes/no).

Table S2. Association of Quintiles of Food-Based DASH Diet Score with incident AAA by sex.*

	Quintiles of Food-Based DASH Diet Score†					
	**Quintile 1: n=2,670**	**Quintile 2: n=3,425**	**Quintile 3: n=1,979**	**Quintile 4: n=2,686**	**Quintile 5: n=2,736**	**P-value for trend‡**
**Male**						
Median Score (Range)	17.0 (8.0-19.0)	22.0 (20.0-23.0)	25.0 (24.0-25.0)	27.0 (26.0- 28.0)	30.0 (29.0-38.0)	
Events	113	113	45	58	42	
Person-years	29,630	33,791	16,765	19,814	15,610	
IR (95% CI)§	3.81 (3.17- 4.59)	3.34 (2.78- 4.02)	2.68 (2.00- 3.60)	2.93 (2.26- 3.79)	2.69 (1.99- 3.64)	
Model 1 HR (95% CI)‖	1 (ref)	0.77 (0.59-1.00)	0.58 (0.41-0.82)	0.59 (0.43-0.81)	0.50 (0.35-0.71)	<0.001
Model 2 HR (95% CI) #	1 (ref)	0.87 (0.66-1.13)	0.72 (0.50-1.03)	0.72 (0.51-1.01)	0.67 (0.46-0.99)	0.016
**Female**						
Median Score (Range)	18.0 (10.0-19.0)	22.0 (20.0- 23.0)	25.0 (24.0- 25.0)	27.0 (26.0- 28.0)	31.0 (29.0- 38.0)	
Events	31	38	16	36	25	
Person-years	20,930	33,372	23,002	34,689	40,380	
IR (95% CI)§	1.48 (1.04- 2.11)	1.14 (0.83- 1.56)	0.70 (0.43- 1.14)	1.04 (0.75- 1.44)	0.62 (0.42- 0.92)	
Model 1 HR (95% CI)‖	1 (ref)	0.71 (0.44-1.15)	0.40 (0.22-0.74)	0.55 (0.34-0.90)	0.30 (0.17-0.52)	<0.001
Model 2 HR (95% CI)#	1 (ref)	0.75 (0.47-1.22)	0.53 (0.29-0.98)	0.82 (0.50-1.37)	0.49 (0.28-0.86)	0.047

AAA, abdominal aortic aneurysm; ARIC, Atherosclerosis Risk in Communities Study; CI, confidence interval; DASH, Dietary Approaches to Stop Hypertension; HR, hazard ratio; IR, incidence rate.

* Incident AAA cases were ascertained from baseline (1987-1989) through December 31, 2011.

† Food consumption (DASH diet score and individual components) was estimated using cumulative average intake. For those who developed AAA or were censored from the analysis before visit 3, food frequency questionnaire data from visit 1 was used. Otherwise, for those who developed AAA or were censored from the analysis after study visit 3, the average of food frequency questionnaire data from visits 1 and 3 was used.

‡ Trend across quintiles was tested using the median value for the DASH diet score within each quintile.

§ IR (incidence rate) expressed as the number of AAA cases per 1,000 person-years with no adjustment for covariates.

‖ Model 1: Adjusted for total energy intake, race-center, and age by the use of restricted cubic splines (with knots at 45, 51, 57, and 63 years of age, representing the 5th, 35th, 65th, and 95th percentiles).

Model 2: Adjusted for variables in model 1 + alcohol intake quintiles (sex specific quintiles of g/week), education (less than high school, high school or equivalent, college or above), household income (<$25,000; $25,000-$49,999; ≥$50,000), smoking status (current smoker, former smoker with ≥20 pack-years, former smoker with <20 pack-years, never smoker), sport-related physical activity index, leisure-time physical activity index, body mass index category (<25, 25-<30, ≥30 kg/m^2), abdominal obesity (waist-to-hip ratio >0.85 for females and >0.90 for males), hypertension (yes/no), diabetes (yes/no), hypercholesterolemia (yes/no), and cardiovascular disease (yes/no).

Table S3. Association of Quintiles of nutrient based DASH Diet Score with Incident AAA.*

	Quintiles of Nutrient-Based DASH Diet Score†					
	**Quintile 1: (n=3222)**	**Quintile 2: (n=3249)**	**Quintile 3: (n=1662)**	**Quintile 4: (n=2745)**	**Quintile 5: (n=2618)**	**P-value for trend‡**
Median (Range)	1.5 (0.0-2.0)	3.0 (2.5-3.0)	3.5 (3.5-3.5)	4.0 (4.0-4.5)	5.5 (5.0-8.5)	
Events	164	136	49	106	62	
Person-years	62,404	63,640	33,584	55,155	53,200	
IR (95% CI)§	2.63 (2.26-3.06)	2.14 (1.81-2.53)	1.46 (1.10-1.93)	1.92 (1.59-2.32)	1.17 (0.91-1.49)	
Model 1 HR (95% CI)‖	1(ref)	0.88 (0.70-1.11)	0.60 (0.44-0.83)	0.80 (0.62-1.03)	0.51 (0.37-0.69)	<0.001
Model 2 HR (95% CI)#	1 (ref)	0.91 (0.72-1.15)	0.60 (0.43-0.83)	0.90 (0.70-1.17)	0.61 (0.45-0.84)	0.006

* Incident AAA cases were ascertained from baseline (1987-1989) through December 31, 2011.

† Food consumption (DASH diet score and individual components) was estimated using cumulative average intake. For those who developed AAA or were censored from the analysis before visit 3, food frequency questionnaire data from visit 1 was used. Otherwise, for those who developed AAA or were censored from the analysis after study visit 3, the average of food frequency questionnaire data from visits 1 and 3 was used.

‡ Trend across quintiles was tested using the median value for the DASH diet score within each quintile.

§ IR (incidence rate) expressed as the number of AAA cases per 1,000 person-years with no adjustment for covariates.

‖ Model 1: Adjusted for sex, total energy intake, race-center, and age by the use of restricted cubic splines (with knots at 45, 51, 57, and 63 years of age, representing the 5th, 35th, 65th, and 95th percentiles).

Model 2: Adjusted for variables in model 1 + alcohol intake quintiles (sex specific quintiles of g/week), education (less than high school, high school or equivalent, college or above), household income (<$25,000; $25,000-$49,999; ≥$50,000), smoking status (current smoker, former smoker with ≥20 pack-years, former smoker with <20 pack-years, never smoker), sport-related physical activity index, leisure-time physical activity index, body mass index category (<25, 25-29, ≥30 kg/m^2), abdominal obesity (waist-to-hip ratio >0.85 for females and >0.90 for males), hypertension (yes/no), diabetes (yes/no), hypercholesterolemia (yes/no), and cardiovascular disease (yes/no).

Table S4. Association of Individual Nutrients of the Nutrient-Based DASH Diet Score with risk for AAA.[*]

	Quintiles of DASH Nutrient Components[†]					P-value for trend[‡]
Component	**Quintile 1**	**Quintile 2**	**Quintile 3**	**Quintile 4**	**Quintile 5**	
**Saturated Fat**						
Median (range), g/d	8 (1-10)	10 (10- 11)	12 (11- 12)	13 (12- 14)	15 (14- 28)	
Events	92	77	91	96	161	
Person-years	53,859	54,738	53,886	54,095	51,406	
IR (95% CI)[§]	1.7 (1.4- 2.1)	1.4 (1.1- 1.8)	1.7 (1.4- 2.1)	1.8 (1.5- 2.2)	3.1 (2.7- 3.7)	
Model 1 HR (95% CI)[‖]	1(ref)	0.81 (0.60-1.10)	0.94 (0.70-1.26)	0.96 (0.72-1.29)	1.66 (1.28-2.15)	<0.001
Model 2 HR (95% CI)[#]	1(ref)	0.84 (0.62-1.13)	1.00 (0.74-1.33)	0.98 (0.73-1.32)	1.50 (1.15-1.96)	0.001
**Total Fat**						
Median (range), g/d	24 (6-27)	29 (27- 31)	33 (31-34)	36 (34-37)	40 (37- 59)	
Events	97	72	96	107	145	
Person-years	53,397	54,892	54,234	54,068	51,392	
IR (95% CI)[§]	1.82(1.49- 2.22)	1.31(1.04- 1.65)	1.77(1.45- 2.16)	1.98(1.64- 2.39)	2.82(2.40- 3.32)	
Model 1 HR (95% CI)[‖]	1(ref)	0.71 (0.52-0.96)	0.91 (0.68-1.20)	0.96 (0.72-1.26)	1.35 (1.04-1.76)	0.002
Model 2 HR (95% CI)[#]	1(ref)	0.73 (0.53-0.99)	0.94 (0.71-1.26)	0.96 (0.73-1.27)	1.23 (0.94-1.61)	0.020
**Cholesterol**						
Median (range), mg/d	98 (4-113)	126 (113-136)	147 (136-158)	171 (158-188)	217 (188-740)	
Events	117	111	86	78	125	
Person-years	53,644	54,585	54,630	54,531	50,593	
IR (95% CI)[§]	2.18 (1.82- 2.61)	2.03 (1.69- 2.45)	1.57 (1.27- 1.94)	1.43 (1.15- 1.79)	2.47 (2.07- 2.94)	
Model 1 HR (95% CI)[‖]	1(ref)	0.95 (0.74-1.24)	0.77 (0.58-1.02)	0.72 (0.54-0.95)	1.20 (0.93-1.55)	0.690
Model 2 HR (95% CI)[#]	1(ref)	0.96 (0.74-1.25)	0.76 (0.57-1.00)	0.71 (0.53-0.95)	1.10 (0.85-1.43)	0.830
**Protein**						
Median (range), g/d	14 (4-15)	16 (15- 17)	18 (17- 19)	20 (19- 21)	23 (21- 39)	
Events	167	96	100	78	76	
Person-years	51,989	53,710	53,862	54,435	53,987	

IR (95% CI)[§]	3.21 (2.76- 3.74)	1.79 (1.46- 2.18)	1.86 (1.53- 2.26)	1.43 (1.15- 1.79)	1.41 (1.12- 1.76)	
Model 1 HR (95% CI)[‖]	1(ref)	0.53 (0.41-0.68)	0.60 (0.47-0.77)	0.48 (0.36-0.63)	0.55 (0.42-0.73)	<0.001
Model 2 HR (95% CI)[#]	1(ref)	0.57 (0.44-0.74)	0.66 (0.51-0.85)	0.56 (0.42-0.74)	0.63 (0.47-0.84)	0.001
**Fiber**						
Median (range), g/d	7 (1-8)	9 (8-10)	11 (10-11)	12 (11-14)	16 (14-39)	
Events	169	109	73	87	79	
Person-years	50,930	53,835	54,658	54,486	54,072	
IR (95% CI)[§]	3.32 (2.85-3.86)	2.02 (1.68-2.44)	1.34 (1.06-1.68)	1.60 (1.29-1.97)	1.46 (1.17-1.82)	
Model 1 HR (95% CI)[‖]	1(ref)	0.61 (0.47-0.77)	0.41 (0.31-0.54)	0.49 (0.37-0.63)	0.45 (0.34-0.60)	<0.001
Model 2 HR (95% CI)[#]	1(ref)	0.67 (0.53-0.86)	0.48 (0.36-0.64)	0.63 (0.48-0.82)	0.58 (0.43-0.78)	<0.001
**Magnesium**						
Median (range), mg/d	117 (40-130)	140 (130-149)	158 (149-167)	177 (167-190)	208 (190-365)	
Events	138	97	85	88	109	
Person-years	51,920	53,585	54,477	54,427	53,573	
IR (95% CI)[§]	2.66 (2.25- 3.14)	1.81 (1.48- 2.21)	1.56 (1.26- 1.93)	1.62 (1.31- 1.99)	2.03 (1.69- 2.45)	
Model 1 HR (95% CI)[‖]	1(ref)	0.69 (0.53-0.89)	0.56 (0.42-0.73)	0.61 (0.46-0.80)	0.83 (0.63-1.09)	0.078
Model 2 HR (95% CI)[#]	1(ref)	0.75 (0.57-0.97)	0.63 (0.48-0.83)	0.66 (0.50-0.87)	0.93 (0.70-1.23)	0.298
**Calcium**						
Median (range), mg/d	230 (77-273)	312 (273-348)	385 (348-425)	471 (425.0-532.1)	631 (532-1790)	
Events	122	98	108	90	99	
Person-years	52,111	53,908	54,261	54,348	53,355	
IR (95% CI)[§]	2.34 (1.96- 2.80)	1.82 (1.49- 2.22)	1.99 (1.65- 2.40)	1.66 (1.35- 2.04)	1.86 (1.52- 2.26)	
Model 1 HR (95% CI)[‖]	1(ref)	0.67 (0.51-0.87)	0.73 (0.56-0.95)	0.63 (0.48-0.84)	0.73 (0.56-0.96)	0.031
Model 2 HR (95% CI)[#]	1(ref)	0.74 (0.56-0.97)	0.87 (0.67-1.13)	0.77 (0.58-1.02)	0.92 (0.70-1.21)	0.641
**Sodium**						
Median (range), mg/d	703 (233-778)	833 (778-878)	920 (878-964)	1,011 (964-1,068)	1,152 (1068-1,997)	
Events	134	96	83	86	118	
Person-years	52,888	54,506	54,116	54160	52,273	
IR (95% CI)[§]	2.53 (2.14- 3.00)	1.76 (1.44- 2.15)	1.53 (1.24- 1.90)	1.59 (1.28- 1.96)	2.26 (1.88- 2.70)	

Model 1 HR (95% CI)[‖]	1(ref)	0.64 (0.49-0.83)	0.55 (0.42-0.72)	0.55 (0.42-0.72)	0.79 (0.61-1.02)	0.033
Model 2 HR (95% CI)[#]	1(ref)	0.69 (0.53-0.90)	0.60 (0.46-0.79)	0.59 (0.44-0.77)	0.78 (0.60-1.02)	0.033
**Potassium**						
Median (range), mg/d	1,217 (310-1,362)	1,474 (1,362-1,571)	1,662 (1,571-1,752)	1,860 (1752.5-1991.5)	2,172 (1,992-3,725)	
Events	135	105	85	93	99	
Person-years	51,414	53,713	54508	54,513	53,834	
IR (95% CI)[§]	2.63 (2.22- 3.11)	1.95 (1.61- 2.37)	1.56 (1.26- 1.93)	1.71 (1.39- 2.09)	1.84 (1.51- 2.24)	
Model 1 HR (95% CI)[‖]	1(ref)	0.70 (0.54-0.91)	0.57 (0.43-0.75)	0.67 (0.51-0.89)	0.81 (0.61-1.07)	0.101
Model 2 HR (95% CI)[#]	1(ref)	0.73 (0.57-0.95)	0.66 (0.49-0.87)	0.75 (0.56-0.99)	0.91 (0.68-1.22)	0.494
**Sodium/Potassium Ratio**[**]						
Median (range)	0.4 (0.1-0.5)	0.5 (0.5-0.5)	0.6 (0.5-0.6)	0.6 (0.6-0.7)	0.8 (0.7-2.0)	
Events	118	79	91	95	134	
Person-years	53,802	54,383	54,506	54,143	51,148	
IR (95% CI)[§]	2.19 (1.83- 2.63)	1.45 (1.17- 1.81)	1.67(1.36- 2.05)	1.75 (1.43- 2.15)	2.62 (2.21- 3.10)	
Model 1 HR (95% CI)[‖]	1(ref)	0.60 (0.45-0.79)	0.64 (0.49-0.84)	0.64 (0.49-0.84)	0.94 (0.73-1.21)	0.992
Model 2 HR (95% CI)[#]	1(ref)	0.61 (0.45-0.81)	0.66 (0.50-0.87)	0.63 (0.48-0.83)	0.86 (0.66-1.12)	0.409

AAA, abdominal aortic aneurysm; ARIC, Atherosclerosis Risk in Communities Study; CI, confidence interval; DASH, Dietary Approaches to Stop Hypertension; HR, hazard ratio; IR, incidence rate.

[*] Incident AAA cases were ascertained from baseline (1987-1989) through December 31, 2011.

[†] Nutrient consumption was estimated using cumulative average intake. For those who developed AAA or were censored from the analysis before visit 3, food frequency questionnaire data from visit 1 was used. Otherwise, for those who developed AAA or were censored from the analysis after study visit 3, the average of food frequency questionnaire data from visits 1 and 3 was used.

[‡] Trend across quintiles was tested using the median value for the component of the DASH diet score within each quintile.

[§] IR (incidence rate) expressed as the number of AAA cases per 1,000 person-years with no adjustment for covariates.

[‖] Model 1: Adjusted for sex, total energy intake, race-center, and age by the use of restricted cubic splines (with knots at 45, 51, 57, and 63 years of age, representing the 5th, 35th, 65th, and 95th percentiles).

[#] Model 2: Adjusted for variables in model 1 + alcohol intake quintiles (sex specific quintiles of g/week), education (less than high school, high school or equivalent, college or above), household income (<$25,000; $25,000-$49,999; ≥$50,000), smoking status (current smoker, former smoker with ≥20 pack-years, former smoker with <20 pack-years, never smoker), sport-related physical activity index, leisure-time physical activity index, body mass index category (<25, 25-29, ≥30 kg/m^2), abdominal obesity (waist-to-hip ratio >0.85 for females and >0.90 for males), hypertension (yes/no), diabetes (yes/no), hypercholesterolemia (yes/no), and cardiovascular disease (yes/no).

[**] Sodium/Potassium Ratio was defined as (Na mg/1000 kcal)/(K mg/1000 kcal).
